# Adipocentric Strategy for the Treatment of Type 2 Diabetes Mellitus

**DOI:** 10.3390/jcm14030678

**Published:** 2025-01-21

**Authors:** Juan J. Gorgojo-Martínez

**Affiliations:** Department of Endocrinology and Nutrition, Hospital Universitario Fundación Alcorcón, C/Budapest 1, 28922 Alcorcón, Spain; juanjose.gorgojo@salud.madrid.org

**Keywords:** SGLT-2 inhibitor, GLP-1 receptor agonist, tirzepatide, metabolic surgery, adipocentric strategy, adiposopathy-related diabetes

## Abstract

The global prevalence of obesity and type 2 diabetes mellitus (T2D) has risen in parallel over recent decades. Most individuals diagnosed with T2D exhibit adiposopathy-related diabetes (ARD), a condition characterized by hyperglycemia accompanied by three core features: increased ectopic and visceral fat deposition, dysregulated adipokine secretion favoring a pro-inflammatory state, and insulin resistance. Despite advancements in precision medicine, international guidelines for T2D continue to prioritize individualized therapeutic approaches focused on glycemic control and complications, and many healthcare providers predominantly maintain a glucocentric strategy. This review advocates for an adipocentric treatment paradigm for most individuals with T2D, emphasizing the importance of prioritizing weight loss and visceral fat reduction as key drivers of therapeutic intensification. By combining lifestyle modifications with pharmacological agents that promote weight loss—including SGLT-2 inhibitors, GLP-1 receptor agonists, or dual GLP-1/GIP receptor agonists—and, when appropriate, metabolic surgery, this approach offers the potential for disease remission in patients with shorter disease duration. For others, it enables superior metabolic control compared to traditional glucose-centered strategies while simultaneously delivering cardiovascular and renal benefits. In conclusion, an adipocentric treatment framework for ARD, which represents the majority of T2D cases, effectively integrates glucocentric and cardio-nephrocentric goals. This approach constitutes the optimal strategy for ARD due to its efficacy in achieving disease remission, improving metabolic control, addressing obesity-related comorbidities, and reducing cardiovascular and renal morbidity and mortality.

## 1. Introduction: The Crisis of the Glucocentric Model

In recent decades, the prevalence of two chronic and heterogeneous conditions—obesity and type 2 diabetes mellitus (T2D)—has increased in parallel [1,2,3]. This trend is unsurprising given that visceral fat accumulation is the leading risk factor for developing T2D in genetically predisposed individuals [4,5]. According to the CDC’s National Diabetes Statistics Report, 89.8% of American adults with diabetes are overweight or have obesity (body mass index [BMI] > 25 kg/m^2^), and 47.1% meet the criteria for obesity (BMI > 30 kg/m^2^) [6]. Similar findings have been reported in Spain, where 85.3% and 44.9% of patients with T2D have a BMI above 25 kg/m^2^ and 30 kg/m^2^, respectively [7]. However, these figures likely underestimate the true prevalence of excessive body fat among individuals with T2D, as BMI is a suboptimal indicator of adiposity [5,8,9]. An analysis of the NHANES III study showed that the population-level increase in BMI and waist circumference (WC) accounts for 72% of the rise in diabetes prevalence in the United States [10].

Despite advances in precision medicine, the diagnostic criteria for obesity and T2D remain imprecise. Most guidelines continue to use BMI for diagnosing obesity, despite its limitations as a marker of cardiometabolic risk, total body fat, and, critically, visceral and ectopic fat [5]. Measures such as WC correlate more strongly with abdominal visceral fat and its associated risks [5]. On the other hand, the diagnosis of T2D, despite accounting for around 90% of diabetes cases, remains a diagnosis of exclusion [11], as it requires ruling out autoimmune diabetes, monogenic diabetes, and other less common types of diabetes described in the ADA classification [12]. Moreover, its definition is highly imprecise, encompassing individuals with “relative insulin deficiency and insulin resistance”, most of whom have overweight or obesity. It is currently known that T2D cannot be considered a single entity since this term encompasses different types of diabetes with distinct pathogenic mechanisms [13]. While T2D itself is a heterogeneous condition, the predominant subtype—driven by visceral and ectopic fat accumulation—represents a relatively homogeneous entity in genetically diverse individuals [14].

These diagnostic shortcomings have contributed to the widespread adoption of a glucocentric therapeutic strategy for all patients with T2D. While intensive glycemic control is effective to reduce microvascular complications, its impact on cardiovascular (CV) morbidity and mortality remains less well-established. Nonetheless, the glucocentric approach has dominated treatment guidelines for decades [15].

A pivotal shift occurred during 2007–2008, when evidence emerged challenging the intensive glucocentric model and leading to revised regulatory standards for antihyperglycemic therapies, which now require evidence of CV safety. Firstly, rosiglitazone, a PPARγ receptor agonist, had demonstrated greater durability in glycemic control compared to metformin or sulfonylureas in the ADOPT study [16]. However, results from a meta-analysis showed that this drug appeared to increase the risk of myocardial infarction, thereby decoupling the concepts of glycemic control and CV protection [17]. Besides that, the ACCORD trial demonstrated that intensive glycemic control failed to reduce CV morbidity and mortality and was linked to increased all-cause mortality [18]. Weight gain and hypoglycemia in the intensive-treatment groups might have contributed to these findings, though causal relationships remain unproven. Meta-analyses suggest that while intensive hyperglycemia treatment modestly reduces non-fatal myocardial infarctions, it has no significant impact on mortality, stroke, or heart failure [19].

In 2008, the FDA established regulatory criteria requiring meta-analyses or dedicated clinical trials to prove the CV safety of new antihyperglycemic drugs prior to approval. Thanks to this initiative, it was demonstrated that novel drugs such as several glucagon-like peptide 1 receptor agonists (GLP-1RAs) and sodium-glucose co-transporter-2 inhibitors (SGLT-2is) not only improved glycemic control without causing hypoglycemia but also promoted weight loss and reduced CV and renal morbidity and mortality.

Despite these advances, metformin has remained a first-line therapy in many glucocentric algorithms [20] largely due to its antihyperglycemic efficacy, affordability, weight-neutral effects, and the reduction in CV morbidity and mortality observed in a small group of newly diagnosed overweight T2D patients with low CV risk in the UKPDS study when compared to diet, sulfonylureas, or first-generation insulins [21]. However, these findings would not meet modern regulatory standards due to significant methodological limitations, including sample size, lack of blinding, and exclusion of high CV risk patients. Indeed, a meta-analysis of randomized trials concluded that metformin has a neutral effect on the incidence of myocardial infarction and CV or all-cause morbidity and mortality [22]. Consequently, recent trials demonstrating the CV and renal benefits of GLP-1RAs and SGLT-2is have progressively displaced metformin from its longstanding position as the first-line therapy in several T2D treatment algorithms [23,24].

Over the past 15 years, various authors have advocated for a paradigm shift from the classic glucocentric model to a weight-centric approach [15,25,26,27,28]. Weight loss, particularly targeting dysfunctional visceral and ectopic fat, provides numerous benefits for T2D patients, including improved glycemic control, better management of CV risk factors, diabetes remission, prevention of microvascular and macrovascular complications, and reduced mortality [27]. However, current weight-centric approaches often rely on BMI, which inadequately reflects visceral adiposity [5,29]. This limitation risks excluding T2D patients with normal BMI but pathological increases in body fat. Studies have demonstrated a dose–response relationship between WC and all-cause mortality, even in individuals with normal BMI, highlighting the need to evaluate fat distribution rather than relying solely on BMI [29].

A new therapeutic paradigm—termed adipocentric—is, therefore, necessary [30]. This approach begins with a differential diagnosis to identify a specific subset of patients with T2D characterized by increased visceral and ectopic adiposity, often referred to as “diabesity” [31] or, more appropriately, “adiposopathy-related diabetes” (ARD). For these patients, treatment should prioritize weight loss targeting dysfunctional fat through lifestyle interventions, pharmacologic agents, or metabolic surgery (MS). This strategy aims to achieve optimal glycemic control, reduce CV morbidity and mortality, and lower all-cause mortality. Simultaneously, the model must recognize less common diabetes subtypes unrelated to adiposopathy and apply alternative therapeutic approaches for these patients.

## 2. Adiposopathy as the Most Frequent Cause of T2D

The percentage of body fat in a healthy adult is below 20% in men and 30% in women. Thresholds for obesity have been proposed as a body fat percentage above 25% and 35% in adult men and women, respectively [32]. More than 80% of white adipose tissue is subcutaneous, primarily located in the abdominal and gluteofemoral regions. Visceral adipose tissue accounts for the remaining 10–20% of total body fat in men and 5–10% in women. Additionally, small deposits of brown adipocytes are present in regions such as the supraclavicular, paravertebral, and mediastinal areas. These brown adipocytes can be activated to generate heat in response to cold exposure, with their activity inversely correlated with age, BMI, and fasting glucose levels [33]. Beige adipocytes, scattered within white adipose tissue, can convert into brown adipocytes in response to cold exposure, exercise, and endocrine signals [33].

The genome of *Homo sapiens sapiens* is evolutionarily adapted to episodic food intake and prolonged fasting, a hallmark of prehistoric times [34]. In the modern era, humans face an excess caloric supply without a compensatory increase in energy expenditure. This disruption of energy balance, induced by the contemporary obesogenic environment, interacts with common genetic and epigenetic predispositions for central obesity and T2D [33], resulting in a pathological response of adipose tissue to positive caloric balance in susceptible individuals called adiposopathy. This condition directly and indirectly contributes to the development of diabetes, metabolic syndrome, and CV disease [35].

### 2.1. Central Features of Adiposopathy

The three central features of adiposopathy are as follows [30]:

(a) Increased ectopic and visceral fat: Abnormal accumulation of white adipose tissue in non-physiological locations (e.g., liver, pancreas, heart, and skeletal muscle) and within the visceral compartment (intra-abdominal and retroperitoneal fat).
(b) Adipokine imbalance: A shift towards a pro-inflammatory profile of cytokines produced by adipose tissue.
(c) Insulin resistance.

Thus, the presence or absence of adiposopathy helps explain the heterogeneity of obesity and its manifestations, as the pathogenic potential of excess body fat is determined by adipose tissue dysfunction and ectopic fat deposition rather than simply by increased fat mass [35]. Notably, genetic variants linked to mild lipodystrophy in the general population are associated with higher metabolic and CV risk, lower BMI, and increased visceral-to-subcutaneous adipose tissue ratios [36]. Epidemiological evidence consistently demonstrates that waist circumference (WC) surpasses BMI in predicting CV disease risk [29,37].

In genetically predisposed individuals with skeletal muscle insulin resistance, sustained caloric excess cannot be stored as muscle glycogen but is instead stored as body fat [14]. When subcutaneous white adipose tissue becomes saturated or dysfunctional, excess triglycerides are deposited in atypical sites where fat accumulation is normally minimal—such as the liver, pancreas, heart, blood vessels, kidneys, skeletal muscle, and around the viscera—predisposing an individual to cardiometabolic dysregulation [38]. This excessive ectopic fat deposition is pathologically linked to insulin resistance, metabolic syndrome, systemic inflammation, and CV disease. Because most visceral adipose tissue drains through the portal vein, increased lipolysis of hypertrophic adipocytes exposes the liver to high concentrations of free fatty acids and glycerol, leading to various hepatic metabolic alterations. These include reduced hepatic insulin clearance and higher hyperinsulinemia; de novo lipogenesis with palmitic acid synthesis and increased production of triglyceride-rich lipoproteins, as well as heightened gluconeogenesis [5]. The rise in circulating triglyceride-rich lipoproteins promotes pancreatic steatosis in the context of saturated subcutaneous adipose tissue, contributing to beta-cell dysfunction through lipotoxicity, particularly mediated by palmitic acid [14]. This process is termed the twin-cycle hypothesis (hepatic/pancreatic), which some authors consider the primary mechanism in T2D development.

Conversely, peripheral adiposity, characterized by preferential fat storage in the lower body, may act as a metabolic buffer, mitigating the adverse effects of fat excess [39]. The physiological role of subcutaneous adipose tissue as a metabolic sink for excess triglycerides has limitations due to its finite expansion capacity. When unable to expand through preadipocyte hyperplasia to accommodate positive caloric balance, dysfunctional adipocyte hypertrophy occurs, leading to visceral and ectopic fat deposition. A genetically determined personal fat threshold appears to exist; exceeding this threshold increases the likelihood of developing metabolic disorders such as T2D, even when overall BMI is within the normal range [5,40]. The most extreme example of this threshold is total lipodystrophy, in which there is no capacity to store fat in subcutaneous adipose tissue [14].

Given BMI’s limitations as an individual adiposity indicator, complementary anthropometric measures and body composition techniques are recommended. These approaches reveal distinct obesity phenotypes with varying cardiometabolic risk profiles [41] (Table 1). Among individuals with increased visceral fat, intrahepatic fat shows the strongest association with T2D development, supporting the twin-cycle hypothesis [42,43]. The concept of the metabolically healthy person with obesity has, however, been questioned by several researchers. In an epidemiological study of the UK-based THIN database, which included 3.5 million individuals, people with obesity without metabolic alterations had an increased risk of coronary heart disease, cerebrovascular disease, and HF compared to individuals with normal weight and no metabolic alterations [44]. The main limitation of the study is that the diagnosis of obesity was based exclusively on BMI and not on other surrogate indicators of visceral fat, such as WC, or direct measurements of body composition.

### 2.2. Adipose Tissue as an Active Endocrine Organ

Adipose tissue is not merely a passive fat storage site but an active endocrine organ capable of synthesizing and releasing various bioactive compounds (hormones, chemokines, and cytokines) into circulation, collectively termed adipokines. These affect energy balance, immune responses, vascular homeostasis, angiogenesis, insulin sensitivity, and lipid and carbohydrate metabolism. Dysregulation of adipokines, defined as an imbalance favoring pro-inflammatory over anti-inflammatory compounds, is a hallmark of dysfunctional fat. Pro-inflammatory adipokines, such as leptin, resistin, visfatin, tumor necrosis factor-alpha (TNF-α), IL-1beta, IL-6, and IL-8, among others, increase insulin resistance and exert atherogenic effects. In contrast, adiponectin and IL-10 are considered anti-inflammatory and anti-atherogenic adipokines, whose levels are reduced in visceral obesity [30,38,39].

### 2.3. Inflammation

In obesity, visceral adipose tissue exhibits elevated levels of pro-inflammatory M1 macrophages compared to subcutaneous fat. Monocyte/macrophage infiltration and activation in adipose tissue are likely initiated by increased adipocyte size, induction, and secretion of chemokines such as MCP-1, which are linked to altered angiogenesis, abnormal vascular development, and increased fibrosis. Infiltrating inflammatory macrophages are the main source of pro-inflammatory mediators, especially TNF-α, within adipose tissue and likely synergize with adipocytes to amplify local inflammation. Hypoxia, fibrosis, and mitochondrial dysfunction in adipose tissue also contribute to diabetes development [33].

### 2.4. Visceral Obesity, Lipotoxicity, and Beta-Cell Dysfunction

Elevated levels of triglyceride-rich lipoproteins (termed lipotoxicity), pro-thrombotic factors, and inflammatory cytokines in visceral obesity contribute to insulin resistance and glucotoxicity. Along with chronic sympathetic nervous system hyperactivity, this induces metabolic syndrome, accelerating beta-cell dysfunction in genetically predisposed individuals and leading to diabetes development [33,45]. Beta-cell dysfunction involves secretory impairment, apoptosis, and dedifferentiation, although beta-cell dedifferentiation may be reversible with appropriate interventions [14].

## 3. Heterogeneity vs. Homogeneity in the Pathophysiology of T2D

T2D is widely considered a multifactorial disease, exhibiting substantial heterogeneity in the implicated pathophysiological processes, as noted by several authors [13,46,47]. Currently, approximately 400 genetic variants associated with T2D and 12 pathogenic pathways of hyperglycemia are known, many of which contribute to beta-cell dysfunction, the final common denominator of all forms of diabetes. These pathways include defects in pancreatic alpha and beta cells, amyloid accumulation in the pancreas, insulin resistance in the liver, muscle, and adipose tissue, reduced incretin effect, altered central nervous system regulation of glucose metabolism, gut microbiota alterations, systemic inflammation, immune dysregulation, impaired gastric emptying and/or intestinal glucose absorption due to amylin deficiency, and increased renal glucose reabsorption [13].

Acknowledging this multifactorial nature in the pathogenesis of T2D has significant clinical and therapeutic implications. First, treatment should target the patient’s specific pathogenic abnormalities rather than focusing solely on reducing HbA1c levels. Additionally, since no antihyperglycemic medication addresses all pathophysiological defects, combination therapies are almost always required to achieve and maintain long-term glycemic control. Such treatments should be initiated early in the disease course to prevent progressive beta-cell deterioration and should promote weight loss and improvement of other CV risk factors without increasing the risk of hypoglycemia. Ultimately, this tailored approach should translate into better outcomes, including reduced CV and renal morbidity and mortality [25].

The heterogeneity of T2D has inspired efforts to differentiate patients based on underlying disease mechanisms. A notable example is the phenotypic classification proposed by Ahlqvist et al. in Sweden through the ANDIS study, which has been replicated in other populations. This classification divides patients into five groups or clusters based on six clinical variables: age at diagnosis, BMI, HbA1c, anti-GAD antibodies, HOMA-B, and HOMA-R [48,49] (Table 2). However, this system is limited by its high dependence on the timing of diagnosis [14]. For instance, a quarter of individuals may shift phenotypic clusters over five years. Furthermore, the classification relies on BMI, which, as repeatedly highlighted, is an unreliable measure of dysfunctional adiposity. It also fails to identify patients with monogenic diabetes or other secondary causes of T2D, whose phenotypes often overlap with those of T2D. For example, approximately 5% of patients diagnosed with T2D actually have monogenic diabetes [50,51].

To address the challenge of phenotypic variability over the disease course, Udler et al. proposed a genetic-based classification. This system uses 94 polymorphisms and 47 metabolic variables to define five genetic clusters [52]. The first two clusters (“beta-cell” and “proinsulin” clusters) are associated with beta-cell dysfunction, while the remaining three clusters (“obesity”, “lipodystrophy-like”, and “disrupted liver lipid metabolism”) are characterized by insulin resistance. Interestingly, only the “obesity” cluster is directly linked to obesity.

Recent reviews of evidence related to T2D subclassifications conclude that their clinical applicability remains limited. This limitation is largely due to insufficient discriminatory power at the individual level and challenges in implementing these classifications in routine clinical practice [11,53].

In contrast, other authors argue for a pathophysiologically homogeneous view of T2D in genetically heterogeneous individuals [14]. According to this perspective, individuals with T2D possess a genetic predisposition through susceptibility genes that impair beta-cell function, skeletal muscle insulin sensitivity, and subcutaneous adipose tissue expandability, independent of BMI. In these individuals, a positive caloric balance leads to subcutaneous adipose tissue saturation and ectopic fat deposition in the liver and pancreas, triggering the “twin cycle” that drives T2D. The 12 described pathophysiological alterations appear secondarily once diabetes is established and are potentially reversible if the primary pathogenic twin cycle is interrupted. This conceptualization supports a unified treatment strategy for most patients currently diagnosed with T2D, who could be reclassified as having ARD. The therapeutic focus would shift to weight loss with the aim of normalizing hepatic and pancreatic fat content [54].

## 4. Diagnosis of ARD

The previously discussed limitations of using BMI to assess adiposity necessitate expanding the evaluation of patients with diabetes through additional indices and complementary tests. Among anthropometric measures, WC is the most widely used. WC has variable cut-off points based on sex and ethnicity and correlates positively with visceral fat content [5]. Other measures, such as the waist-to-hip ratio and waist-to-height ratio, have been proposed. However, these indices have not achieved the same level of acceptance and consensus as WC.

In recent years, morpho-functional assessment—initially developed for evaluating malnourished patients—has gained prominence due to its diagnostic and prognostic utility in metabolic diseases [55,56,57]. This approach involves a set of techniques designed to evaluate not only total fat content but also adipose tissue dysfunction and distribution, muscle mass, and cellular health. The most commonly used method today is bioelectrical impedance analysis (BIA), which enables bioelectrical impedance vector analysis (BIVA). This technique evaluates raw electrical parameters such as resistance and reactance to derive phase angle and body cell mass. BIA provides information on total body water, fat mass, muscle mass, and phase angle, and it presents these metrics graphically in a coordinate system. A low phase angle may serve as a prognostic marker in various comorbidities associated with obesity [56].

Other, less commonly utilized techniques for body composition assessment in clinical practice include air displacement plethysmography and dual-energy X-ray absorptiometry (DEXA). Computed tomography (CT) and magnetic resonance imaging (MRI) can also measure subcutaneous and visceral fat compartments. However, their applicability in routine clinical settings is highly limited [5]. In this context, nutritional ultrasound has emerged as a promising imaging technique. It evaluates both the size and structure of muscles and adipose compartments, including the subcutaneous and visceral compartments, with a particular focus on the preperitoneal region [55].

Functional tests, such as handgrip dynamometry, are available for assessing muscle strength. Reduced muscle strength is a hallmark of sarcopenic obesity and is associated with increased morbidity and mortality, diminished quality of life, and functional impairments [58].

Lastly, laboratory tests are especially valuable for ruling out other forms of diabetes unrelated to adiposopathy and for assessing various components of the metabolic syndrome. A summary of diagnostic and monitoring tools available for clinical use is provided in Table 3.

## 5. Importance of Weight Loss in the Remission of ARD

The term “remission”, borrowed by diabetologists from oncology, is considered more appropriate than alternatives such as “resolution”, “reversal”, or “cure”. Remission indicates that T2D is neither active nor progressive at that moment but that the individual requires ongoing monitoring due to the potential for disease relapse during follow-up [59]. The definition implies that the individual is not taking medications with clinically relevant antihyperglycemic effects, and at least three months have passed since the discontinuation of such medications or bariatric surgery (BS), as HbA1c reflects glycemic control over the preceding three months [60,61]. The clinical significance of achieving diabetes remission was underscored in the long-term follow-up of patients in the LOOK-AHEAD study, where remission was associated with a 33% reduction in chronic kidney disease (CKD) incidence and a 40% reduction in CV disease compared to those who did not achieve remission [62].

If the twin hepatic/pancreatic cycle hypothesis is correct, weight loss interventions that reduce fat deposits in the liver and pancreas could reverse the pathogenic process and even achieve diabetes remission [54]. In the COUNTERPOINT study, 11 individuals with T2D were evaluated after eight weeks of a very low-calorie diet (600 kcal/day) [63]. This intervention normalized beta-cell function and hepatic insulin sensitivity, which was associated with a decrease in pancreatic and hepatic triglyceride stores. The DIRECT study in the UK randomized 306 individuals with T2D (duration < 6 years, no insulin therapy, BMI 27–45 kg/m^2^) to usual care for T2D or intervention through caloric restriction (approximately 850 kcal/day) for 3–5 months using commercial formulas, followed by a gradual reintroduction of a conventional hypocaloric diet [64]. The goals were a weight reduction higher than 15 kg and T2D remission. After 12 months, 24% of the intervention group versus 0% of the control group had lost more than 15 kg, while 46% versus 4% achieved T2D remission (*p* < 0.0001). This remission percentage increased with greater weight loss, reaching 86% among patients who lost more than 15 kg. At two years of follow-up, the remission rate dropped to 36% [65]. DIRECT confirmed that weight loss reduced de novo lipogenesis to normal levels, as well as intrapancreatic fat content [66]. The total mass of functional beta cells gradually returned to rates similar to those of a matched non-diabetic control group over 12 months and remained stable at 24 months. The DIRECT study results have been replicated in other studies with different populations and similar designs, even among individuals with BMI below 27 kg/m^2^ [14,67,68].

A five-year extension of the DIRECT study involving 85 participants revealed partial weight regain, with a mean sustained weight loss of 6.1 kg and a decline in the remission rate to 13% [69]. These results highlight the challenges of long-term weight maintenance and support the use of medications to sustain weight loss and achieve “drug-induced remission”.

Currently, three drug classes approved for T2D treatment exhibit high antihyperglycemic efficacy and promote weight and visceral fat loss: SGLT-2is, GLP-1RAs, and the dual GLP-1/GIP agonist tirzepatide [70,71,72,73,74,75]. Based on the HbA1c threshold included in the definition of diabetes remission (HbA1c below 6.5%), 53% and 24% of patients assigned to semaglutide 1 mg and canagliflozin 300 mg achieved this target in the SUSTAIN 8 study [71], 67.5% with semaglutide 2.4 mg in the STEP-2 trial [74], and 80% with 10–15 mg tirzepatide in SURMOUNT-2 [75]. Tirzepatide also enabled a significant percentage of patients to achieve normoglycemia, with 43–62% attaining HbA1c below 5.7% in the SURPASS program [76].

MS is recommended for patients with T2D and BMI above 30 kg/m^2^ who have not achieved glycemic or weight loss goals through lifestyle and pharmacological interventions [23,77,78]. Randomized controlled trials have demonstrated higher remission rates with MS vs. standard medical care, particularly with malabsorptive techniques like gastric bypass and biliopancreatic diversion [79,80,81,82,83,84]. For example, in one study, 95% of patients undergoing biliopancreatic diversion and 75% with gastric bypass achieved remission at 5 years, compared to 0% in the medical treatment group [85]. At 10 years, these rates declined to 50% and 25%, respectively. Data from the Swedish Obese Subjects (SOS) study over 15 years [86] and meta-analyses [82] report long-term remission rates of ~30%, with MS, alongside reductions in micro- and macrovascular complications and all-cause mortality.

Baseline predictors of remission after surgery include beta-cell health markers such as age, T2D duration, insulin use, C-peptide levels, and HbA1c [79]. Mechanisms underlying remission through surgery are multifactorial, including weight loss, enhanced nutrient delivery to the distal intestine, resolution of hepatic and pancreatic steatosis, increased anorexigenic gut hormone secretion, altered bile acid circulation, and gut microbiota changes [87,88].

Patients achieving remission through weight loss or surgery remain in a “post-diabetes” state requiring vigilance due to relapse risk, persistent elevated CV risk, and potential long-term complications from glycemic memory effects. For patients failing to achieve remission or experiencing relapse, chronic antihyperglycemic treatment should prioritize weight loss. Ethical dilemmas arise regarding the discontinuation of CV and renal-protective therapies like GLP-1RAs and SGLT-2is after MS in high CV-renal risk individuals. To address this, the concept of “drug-induced” or “drug-sensitive” remission has emerged, where normoglycemia is maintained with antihyperglycemic agents mimicking caloric restriction, such as GLP-1RAs, dual GLP-1/GIP agonists, and SGLT-2is [61].

## 6. Importance of Weight Loss in Metabolic Control and Cardiorenal Protection in Patients with ARD

As discussed in the previous section, weight loss achieved through caloric restriction—via lifestyle modification, pharmacotherapy, or bariatric surgery—reduces intrahepatic fat, insulin resistance, hepatic glucose production, circulating triglycerides, and their accumulation in pancreatic islets. The resolution of pancreatic steatosis fosters the redifferentiation of beta cells, which were dedifferentiated but not dead due to fat accumulation, leading to the normalization of pancreatic endocrine function. These effects are most pronounced with weight loss exceeding 15% of baseline body weight [66]. Moderate weight losses (5–15%) reduce glucolipotoxicity, thereby improving insulin sensitivity, glycemic control, blood pressure, lipid profiles, and reducing the need for antihyperglycemic medications [14,23,27].

Growing evidence underscores the broader benefits of weight reduction. A body weight loss of more than 10% has been shown to ameliorate metabolic comorbidities, reduce CV morbidity and mortality, improve metabolic-associated steatotic liver disease, alleviate sleep apnea, and enhance overall quality of life [23]. For instance, a subanalysis of the LOOK-AHEAD study, which did not achieve primary CV superiority, revealed that T2D patients who lost more than 10% of their baseline weight in the first year experienced a significant 21% reduction in CV morbidity and mortality compared to those who did not lose weight [89]. Similarly, a meta-analysis demonstrated that MS in patients with T2D significantly reduced microvascular and macrovascular complications and all-cause mortality compared to non-surgical treatments [82].

The general target for patients with ARD should be a weight loss of at least 10–15% within 6–12 months of intervention. This should be accompanied by reductions in WC and fat mass, as assessed if available through body composition analysis, while preserving muscle mass and function. After reaching a weight-loss plateau, the impact on diabetes and other cardiometabolic risk factors should be re-evaluated. If control targets are not achieved, treatment should be intensified to facilitate further weight loss [23,27].

### 6.1. Lifestyle Modification

Patients should be enrolled in a structured lifestyle intervention program aimed at weight loss, incorporating a healthy eating plan, physical activity, and behavioral intervention. These programs can be delivered in primary care or specialized care settings, depending on the severity of obesity [23,27]. When feasible, interventions should include high-frequency counseling (at least 16 sessions within six months), focusing on dietary changes, physical activity, and behavioral strategies to create an energy deficit of 500–750 kcal/day [27]. While international guidelines do not prioritize specific dietary patterns for patients with diabetes and overweight/obesity [90], some studies highlight the benefits of a Mediterranean diet, which can be introduced at the outset or following a very-low-calorie diet aimed at achieving diabetes remission [13].

The Mediterranean diet is characterized by a high intake of fruits, vegetables, legumes, whole grains, fish, and unsaturated fats (particularly olive oil), moderate consumption of alcohol (mainly wine, preferably during meals), and low intake of red meat, dairy products, and saturated fats. A meta-analysis demonstrated superior glycemic control with a Mediterranean diet compared to other dietary patterns [91]. In the PREDIMED trial conducted in Spain, 7447 participants at high CV risk (approximately half with T2D) but without established CV disease were randomized to one of three groups: a Mediterranean diet supplemented with extra virgin olive oil, a Mediterranean diet supplemented with nuts, or a low-fat diet recommended by the American Heart Association [92]. After five years, the Mediterranean diet with olive oil reduced CV morbidity and mortality (myocardial infarction, stroke, or CV death) by 31% and the diet with nuts reduced these outcomes by 28%, compared to the low-fat diet. These benefits extended to the T2D subgroup. Additionally, the Mediterranean diet reduced T2D incidence by 52% compared to the low-fat diet. Notably, the PREDIMED diets were ad libitum, with no physical activity promotion or weight loss guidance. The mechanisms behind the association between adherence to the traditional Mediterranean diet and reduced CV risk may include decreased low-grade inflammation, higher adiponectin levels, lower coagulability, improved endothelial function, reduced oxidative stress, lower concentrations of atherogenic lipoproteins, decreased oxidized LDL particles, and reduced macrophage uptake of oxidized LDL [93].

While the PREDIMED study provides valuable insight into the potential benefits of dietary interventions in patients with high CV risk (with and without T2D), its direct applicability to ARD remains uncertain. Nevertheless, it is biologically plausible that similar mechanisms underlying the efficacy of the Mediterranean diet in the PREDIMED population may translate to benefits for individuals with ARD. Further research is warranted to confirm the CV effects of the Mediterranean diet in this specific population. The ongoing PREDIMED-PLUS trial, a multicenter, randomized study of primary CV prevention, includes 6874 participants with a BMI of 27–40 kg/m^2^ and metabolic syndrome [94]. Participants have been randomized to two groups: a control group following a Mediterranean diet supplemented with extra virgin olive oil and nuts without calorie restriction or physical activity guidance, and an intensive intervention group adhering to a hypocaloric Mediterranean diet (30% calorie restriction) supplemented with olive oil and nuts, alongside an intensive lifestyle program promoting physical activity (e.g., 45 min of daily walking) and weight loss goals with behavioral therapy. The primary objective is to demonstrate a reduction in CV morbidity and mortality with the intensive intervention. Weight loss goals include an average reduction in body weight and WC of over 8% and 5%, respectively, within the first six months, sustained over the following 7.5 years.

For patients with overweight or obesity and T2D, a Mediterranean diet similar to that of the PREDIMED study can be recommended, with adaptations from PREDIMED-PLUS to promote weight loss.

Regular moderate-to-intense physical activity offers significant benefits for metabolic control and CV risk factors in T2D [90,95,96]. Intervention programs improve glycemic control, with the most significant effects seen when resistance and aerobic exercises are combined. Higher total physical activity levels are associated with reduced CV and all-cause mortality compared to lower levels.

While physical activity alone has a modest effect on weight loss, when combined with a hypocaloric diet, it enhances fat loss while preserving lean mass. Sustained physical activity helps prevent weight regain. Aerobic and resistance training improve insulin sensitivity, glycemic control, lipid profiles, and blood pressure, supporting weight management efforts. For sedentary individuals, initiating moderate exercise is recommended, with pedometers as a practical tool to track progress toward a goal of 10,000–15,000 steps per day. Guidelines recommend at least 150 min per week of moderate-intensity aerobic exercise, along with two weekly resistance training sessions (8–12 repetitions × 3 sets per muscle group). Additional benefits can be achieved by gradually increasing to 300 min of moderate-intensity aerobic exercise or 150 min of high-intensity exercise weekly. A clinical evaluation, including stress testing, is advisable for sedentary individuals with CV risk factors who plan to engage in high-intensity exercise [97].

### 6.2. Antihyperglycemic Drugs with Weight and Cardiorenal Benefits

In recent years, two therapeutic classes of antihyperglycemic drugs—GLP-1RAs and SGLT-2is—have demonstrated significant efficacy not only in glycemic control but also in promoting weight loss and reducing CV and renal morbidity and mortality in patients with T2D (Table 4) [24,98,99,100,101,102,103,104,105]. These drugs have complementary mechanisms of action and are currently recommended for combined use [70,106,107,108,109,110,111]. Meta-analyses have shown that both classes provide substantial CV and renal protection. GLP-1RAs reduce major adverse CV events (MACE) by 14%, CV death by 13%, all-cause mortality by 12%, myocardial infarction by 10%, stroke by 17%, hospitalizations for HF by 11%, and CKD progression by 21% [98]. SGLT-2is similarly reduce MACE by 12%, CV death by 15%, all-cause mortality by 14%, myocardial infarction by 11%, hospitalizations for HF by 32%, and CKD progression by 36%, although they do not significantly reduce the risk of stroke [99,100].

SGLT-2is are a class of orally administered drugs that induce the elimination of 60–80 g of glucose per day in the urine, with glucosuria reaching up to 120 g/day at high doses of canagliflozin [24,106,107,108]. Unlike other glucose-lowering agents (which are highly specific for SGLT-2), canagliflozin at a dose of 300 mg transiently inhibits SGLT-1 in the intestine (responsible for the absorption of glucose and galactose), reducing postprandial glucose levels and stimulating distal secretion of GLP-1 and PYY, likely due to the metabolism of glucose by the microbiota into short-chain fatty acids and the stimulation of intestinal L cells. This effect may explain differences in efficacy (HbA1c, weight, a d blood pressure [BP]) between canagliflozin 300 mg and other SGLT-2is [112].

Glucosuria associated with SGLT-2is reduces fasting glucose, postprandial glucose, and HbA1c levels while promoting weight loss through calorie excretion, particularly targeting visceral adipose tissue. Compensatory metabolic responses, such as transient increases in glucagon secretion and hepatic glucose production, may occur [24,106,107,108]. Additionally, SGLT-2is induce a pseudo-fasting state that activates nutrient deprivation pathways, autophagy, and cytoprotection via sirtuins and adenosine monophosphate–activated protein kinase (AMPK), leading to increased ketogenesis and hematocrit [113]. Uric acid excretion is facilitated through glucose exchange in the kidney (via GLUT-9), reducing serum uric acid levels. Mild natriuresis and osmotic diuresis (approximately 300 mL/day) further contribute to significant reductions in both systolic and diastolic BP.

The CV benefits of SGLT-2is are attributed to three main mechanisms [107,113]:

1. Metabolic effects: Reduction in glucotoxicity, visceral fat, and uric acid levels;
2. Hemodynamic effects: Lowered preload (volemia) and afterload (BP);
3. Direct myocardial effects: Activation of nutrient deprivation signals, inhibition of the myocardial sodium-hydrogen exchanger type 1 (NHE-1), and reduction of myocardial fibrosis and pro-inflammatory adipokines derived from epicardial and perivascular fat.

Similarly, SGLT-2is exhibit protective effects on renal function through [107,108,113]:

1. Metabolic mechanisms: Reductions in glucotoxicity, peri-/intrarenal fat, and activation of nutrient deprivation pathways;
2. Systemic hemodynamic effects: Decreased systolic BP transmitted to the kidney;
3. Intrarenal hemodynamic effects: Restoration of tubule-glomerular feedback;
4. Tubulointerstitial mechanisms: Reduced glucotoxicity, proteinuria, and activation of anti-inflammatory and antifibrotic pathways.

GLP-1RAs are a pharmacological class of peptides that activate the human GLP-1 receptor [109,110,111]. Activation of this receptor not only lowers blood glucose levels by stimulating insulin secretion from beta cells, but also reduces glucagon secretion from alpha cells through the promotion of paracrine somatostatin secretion from delta cells. Importantly, these drugs do not induce hypoglycemia, as their effects are self-limiting at low plasma glucose levels. The weight and fat loss associated with GLP-1RAs is attributed to their anorexigenic effects, mediated by receptors in various regions of the central nervous system, including the arcuate nucleus of the hypothalamus and the mesolimbic reward circuit.

Unlike SGLT-2is, the risk curves for atherosclerotic CV events with GLP-1RAs diverge slowly. This progressive effect resembles that observed with other cardioprotective drugs such as statins and appears to be mediated by anti-atherosclerotic and anti-inflammatory mechanisms [109,110,111]. GLP-1RAs confer cardioprotection through:

1. Direct mechanisms: Improved coronary vascularization, vasodilation, anti-inflammatory and antioxidant effects, enhanced endothelial function, inhibition of smooth muscle proliferation, plaque stabilization, and increased ischemia tolerance;
2. Indirect mechanisms: Reduced glucotoxicity and lipotoxicity, improved myocardial glucose utilization, lowered BP, and decreased inflamed epicardial fat.

Renal protection with GLP-1RAs involves:

1. Systemic effects: Reduced glucotoxicity, body weight, and systolic BP;
2. Hemodynamic effects: Inhibition of NHE-3 in the proximal tubule, inducing natriuresis and restoring tubule-glomerular feedback;
3. Anti-inflammatory effects: Reduced proteinuria and activation of antifibrotic pathways.

The recent approval of tirzepatide, a dual GLP-1 and GIP receptor agonist, has expanded therapeutic options for T2D and obesity [114,115]. Both GIP and GLP-1 play roles in regulating food intake by stimulating neurons in the brain’s satiety center. They also stimulate insulin secretion from pancreatic beta cells, but their effects on glucagon production by pancreatic alpha cells differ: GIP exerts a glucagonotropic effect during hypoglycemia, while GLP-1 exhibits a glucagonostatic effect during hyperglycemia. Additionally, GIP directly stimulates lipogenesis, whereas GLP-1 indirectly promotes lipolysis, collectively maintaining healthy adipocytes, reducing ectopic fat distribution, and increasing adiponectin production and secretion by adipocytes. Together, these two incretins contribute to metabolic homeostasis, preventing hyperglycemia and hypoglycemia while mitigating dyslipidemia [115]. For some authors, the effect of tirzepatide on GIP receptors remains unclear, as the drug favors their downregulation, acting more like a functional antagonist [116], as evidenced by the greater reduction in plasma glucagon with tirzepatide in T2D patients compared to dulaglutide [117]. At the time of writing, the SURPASS-CVOT CV safety trial comparing tirzepatide to dulaglutide has not yet been completed.

Comparative studies of these three therapeutic families conclude that the dual GLP-1/GIP agonist tirzepatide is currently the most effective drug for glycemic control and weight loss, followed by GLP-1RAs and SGLT-2is [71,118]. All three therapeutic groups have a visceral fat-reducing effect [72,73]. A recent meta-analysis further supports the superiority of GLP-1-based therapies over basal insulin in achieving better glycemic control and weight and BP outcomes [119], underscoring the benefits of an adipocentric pharmacological approach for both glycemic and weight management.

The clinical trials included in this review have been conducted in outpatient settings. Evidence regarding the use of GLP-1 RAs in hospitalized patients remains limited, primarily derived from research studies and selected populations of medically stable individuals. For patients with T2D hospitalized due to HF, it is recommended to initiate or continue treatment with a SGLT2i during hospitalization and upon discharge, provided there are no contraindications, and the patient has recovered from the acute illness [120].

### 6.3. Metabolic Surgery

A prior section reviewed the role of MS in achieving remission of type 2 diabetes (T2D) [79,80,81,82]. However, not all patients achieve remission in the short term, and approximately two-thirds of individuals undergoing surgery—particularly those with more advanced diabetes—experience persistence or recurrence of the disease over the long term [85,86]. Nevertheless, these patients generally exhibit improved glycemic control following the intervention, reduced reliance on antihyperglycemic agents, including insulin, and enhanced blood pressure and lipid profiles. These improvements are associated with a lower risk of microvascular and macrovascular complications, as well as reduced mortality [81,82]. For example, in a randomized clinical trial, insulin use at 10 years was 53.3% in the medical treatment group, 5% in the gastric bypass group, and 0% in the biliopancreatic diversion group [85].

In patients with established CV disease, HF or CKD, continuation of GLP-1RAs and SGLT-2is may be recommended after MS due to their cardio- and nephroprotective effects, regardless of diabetes status. Additionally, in patients who experience persistence or recurrence of diabetes following MS, liraglutide, a GLP-1 RA, demonstrated efficacy and safety in clinical trials and may help prevent long-term weight regain [121,122]. However, no published clinical trials have evaluated the use of SGLT-2is or tirzepatide after MS.

Recent clinical trials and meta-analyses suggest that long-term glycemic and weight outcomes are superior in T2D patients who undergo gastric bypass compared to those who undergo sleeve gastrectomy [83,84,123,124,125]. As a result, gastric bypass is currently considered the preferred surgical technique.

## 7. Proposed Therapeutic Strategy for Patients with ARD

**Figure 1:**
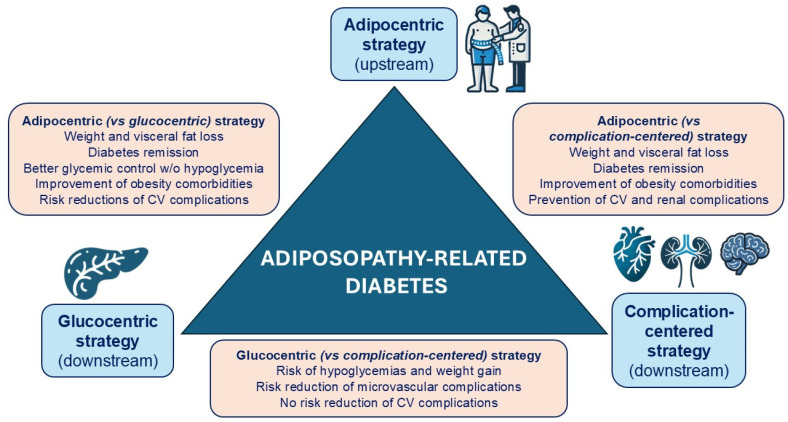
Benefits of an adipocentric approach versus a glucocentric or a complication-centered approach in the management of adiposopathy-related diabetes.

Current international guidelines recommend individualizing therapeutic strategies for patients with T2D based on their disease phenotype. These guidelines advocate for a weight-centered strategy for diabetes associated with excess weight, a glucose-centered strategy for beta-cell dysfunction, and a complication-centered strategy when CV disease, HF, or CKD is present [27,70]. However, after reviewing the available evidence, it is apparent that most patients currently categorized as having T2D actually have ARD. These individuals would benefit from an adipocentric strategy (beyond weight-centric), integrating both glucocentric and cardio-nephrocentric approaches [15,25,28] (Figure 1).

The use of additional indices and diagnostic tools, such as WC, body fat percentage via bioimpedance, metabolic syndrome criteria, or specific tests for fatty liver disease diagnosis, is likely to reduce the proportion of patients without ARD to a small figure [14]. For patients with normal weight and WC, differential diagnosis should include measuring anti-GAD antibodies, stimulated C-peptide, and utilizing online calculators for monogenic diabetes risk (e.g., *diabetesgenes.org*/*exeter-diabetes-app*/*ModyCalculator*) [12]. Autoimmune diabetes should be particularly suspected in individuals under 35 years of age with a BMI below 25 kg/m^2^, a personal or family history of autoimmune diseases (including type 1 diabetes), or treatment with immunomodulatory drugs that could precipitate autoimmune diabetes.

The primary goal of the adipocentric strategy, which will guide treatment intensification decisions, would not be HbA1c but rather achieving a weight loss of at least 10%, preferably 15%, along with a reduction in WC and body fat percentage [14,27]. This degree of weight reduction can lead to disease remission in patients with a shorter disease duration and, in others, to a higher degree of glycemic control than is achieved with the classic glucose-centric strategy, given the superior efficacy of new drugs, alongside CV and renal benefits.

**Figure 2:**
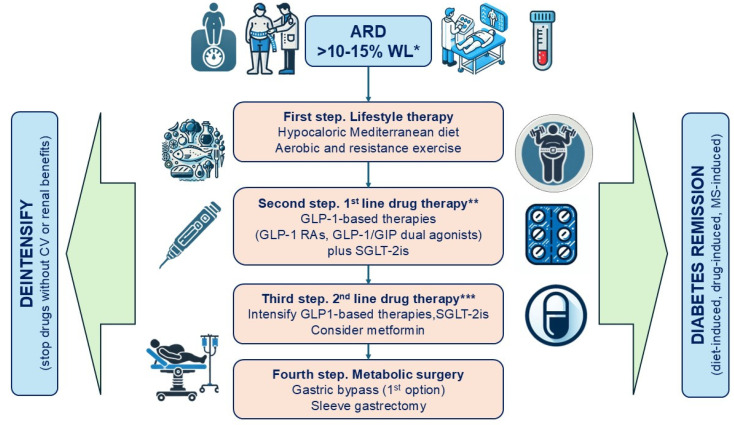
Therapeutic algorithm for the treatment of adiposopathy-related diabetes. * The primary goal of the adipocentric strategy is a weight loss of at least 10%, preferably 15%, along with a reduction in waist circumference and body fat percentage. Additional goals are disease remission or optimal glycemic control. In cases where patients diagnosed with ARD show unfavorable glycemic progression, alternative diabetes etiologies should be suspected. ** For patients with established CV or renal complications, drugs with proven benefits in randomized clinical trials should be prioritized. *** Intensification with GLP-1RAs may involve switching to more effective agents within the class, titrating to higher doses or switching to tirzepatide. For patients on SGLT-2is, intensification may include using the highest dose of canagliflozin (300 mg/day). *ARD*: *adiposopathy-related diabetes*. *MS*: *metabolic surgery*.

Treatment for ARD combines lifestyle modification with weight-loss-promoting drugs such as SGLT-2is and GLP-1-based therapies (monoagonists and dual GLP-1/GIP agonists), either sequentially or simultaneously (Figure 2). BMI and HbA1c levels guide initial treatment intensity, such as using combination pharmacotherapy upfront or selecting the most potent agents [24]. For patients with established CV or renal complications, drugs with proven benefits in randomized clinical trials should be prioritized. Meta-analyses of clinical trials suggest additive CV and renal benefits from combining GLP-1RAs and SGLT-2is [126,127,128].

Therapeutic intensification with GLP-1RAs may involve transitioning to more effective agents within the class, escalating doses (e.g., semaglutide 2.4 mg), or switching to tirzepatide [70]. For patients on SGLT-2is, intensification may include using the highest dose of canagliflozin (300 mg/day), although this approach is supported mainly by observational data [112,129]. Metformin is recommended as third-line therapy if glycemic control targets remain unmet. For patients failing to achieve weight-loss or glycemic goals, MS is indicated, with GLP-1RAs and SGLT-2is potentially continued postoperatively depending on the patient’s progress and clinical profile.

Pharmacological deintensification should be considered for patients reevaluated years after a T2D diagnosis [70]. For those on metformin, it may be maintained due to its benefits for glycemic control without causing weight gain or hypoglycemia. However, weight loss achieved through lifestyle interventions and GLP-1- or SGLT-2-based therapies may permit discontinuation of medications that promote weight gain and/or hypoglycemia (e.g., sulfonylureas, glinides, pioglitazone, and insulin) or that lack CV or renal benefits (all the aforementioned plus DPP-4 inhibitors) [24,70]. For instance, a recent study comparing tirzepatide to preprandial insulin lispro in patients with T2D treated with insulin glargine (mean dose 46 U/day) demonstrated that patients receiving tirzepatide (15 mg dose) ended the study with an average glargine dose of only 8 U/day (versus 112 U/day in the lispro group). This group also achieved better glycemic control, greater weight loss, and fewer hypoglycemic episodes compared to those on basal-bolus insulin therapy [130].

In cases where patients diagnosed with ARD show unfavorable glycemic progression, alternative diabetes etiologies should be suspected. At this point, it may be necessary to revisit or expand the differential diagnosis (Table 3) and reclassify the patient’s diabetes type. This could necessitate specific treatments, such as insulin for autoimmune diabetes or sulfonylureas for certain monogenic diabetes forms [70].

While lifestyle modifications, weight-loss medications, and MS are effective strategies for managing ARD, sustaining long-term weight loss remains a significant challenge. Evidence indicates that a significant proportion of individuals who achieve weight loss through lifestyle modifications eventually regain much, if not all, of the lost weight due to hormonal and metabolic adaptations that promote a positive energy balance [27]. This weight regain can have adverse implications for long-term ARD remission, as the reaccumulation of fat mass may lead to the reactivation of pathogenic mechanisms underlying the disease. These observations highlight the importance of adopting a long-term, multimodal approach to ARD management. Such an approach should include sustained lifestyle support, chronic pharmacotherapy, and ongoing follow-up in patients with and without MS in order to mitigate the risk of weight regain and diabetes relapse.

Social determinants of health, including socioeconomic status, education level, access to healthcare, and systemic inequities, play a pivotal role in the prevalence, progression, and outcomes of obesity and ARD, particularly among marginalized populations. Vulnerable groups such as minoritized racial/ethnic communities and individuals with low income often face compounded barriers to achieving optimal metabolic health due to limited access to nutritious foods, safe spaces for physical activity, and comprehensive medical care [131]. These disparities are further exacerbated by stigmatization of obesity, which undermines trust in healthcare systems and engagement in long-term care. An adipocentric approach to managing ARD provides an opportunity to address these inequities by prioritizing interventions that target the root cause of disease—excess adiposity—through culturally tailored, community-based, and patient-centered strategies. By focusing on underlying mechanisms rather than solely glycemic control, this paradigm has the potential to mitigate the disproportionate burden of ARD among vulnerable populations, improve health outcomes, and advance equity in diabetes care. Efforts should focus on ensuring fair access to effective interventions on ARD to avoid deepening healthcare disparities.

## 8. Conclusions

The recognition that most patients currently diagnosed with T2D actually have diabetes induced by dysfunctional excess body fat invites consideration of a shift in nomenclature, potentially to ARD. At present, diagnostic tools are available to identify the minority of individuals with T2D whose disease etiology differs and who therefore require alternative therapeutic strategies. An adipocentric approach, centered on reducing weight and dysfunctional body fat, represents the optimal strategy for ARD due to its efficacy in achieving disease remission, improving metabolic control, addressing obesity-related comorbidities, and reducing CV and renal morbidity and mortality.

We do not need to choose between a glucocentric, adipocentric, or cardio-nephrocentric strategy. An adipocentric therapeutic algorithm driven by weight loss exceeding 10–15% can effectively integrate all three approaches. In the coming years, the development and commercialization of new oral GLP-1RAs [132], GLP-1RAs in higher-dose formulations [133], dual GLP-1/glucagon agonists [134], triagonists targeting GLP-1/GIP/glucagon pathways [135], and combinations of GLP-1RAs with amylin mimetics [136] are expected to make achieving weight loss levels currently attainable only through MS more feasible.

However, therapeutic inertia, delays in the implementation of guidelines into clinical practice, and socioeconomic and healthcare disparities across different regions of the world [137] are likely to remain significant challenges for these recommendations in the coming years.

## Figures and Tables

**Table 1:** Summary of different obesity phenotypes. BMI: body mass index, MUNW: metabolically unhealthy normal weight, MHO: metabolically healthy overweight/obesity, MUO: metabolically unhealthy overweight/obesity, SO: sarcopenic obesity [41].

	MUNW	MHO	MUO	SO
BMI (kg/m^2^)	Normal	High	High/ Very high	High/ normal
Waist circumference	Normal/ High	Normal	High	High
Metabolic syndrome	Present	Absent	Present	Present
Visceral fat	High	Low	High	High
Lean mass	Normal	High	Normal/ High	Low
Physical performance	Low	High	Low/ Very low	Very low

**Table 2:** Characteristics of diabetes subtypes according to the Ahlqvist model. Refs: [48,49].

**Latent Autoimmune Diabetes in Adults (Severe Autoimmune Diabetes, SAID)** ⚬ Positive for glutamic acid decarboxylase (GAD) antibody ⚬ Early onset ⚬ Low BMI ⚬ Low insulin secretion ⚬ High HbA1c ⚬ Early requirement for insulin therapy ⚬ High risk of retinopathy ⚬ High incidence of nephropathy (dependent on baseline eGFR) **Severe Insulin-Deficient Diabetes (SIDD)** ⚬ GAD antibody-negative but other characteristics similar to SAID ⚬ Low insulin secretion ⚬ High HbA1c ⚬ Early requirement for insulin therapy ⚬ High risk of retinopathy, nephropathy, neuropathy, and erectile dysfunction **Severe Insulin-Resistant Diabetes (SIRD)** ⚬ GAD antibody-negative ⚬ Obesity ⚬ Insulin resistance ⚬ Late onset ⚬ High risk of kidney disease and fatty liver ⚬ High risk of ischemic heart disease and stroke (depending on age and sex) **Mild Obesity-Related Diabetes (MOD)** ⚬ GAD antibody-negative ⚬ Obesity ⚬ Mild insulin resistance ⚬ Early onset ⚬ Intermediate risk of diabetes-related complications **Mild Age-Related Diabetes (MARD)** ⚬ GAD antibody-negative ⚬ Late onset ⚬ High risk of ischemic heart disease and stroke (depending on age and sex)

**Table 3:** Diagnostic tests in the evaluation of patients with adiposopathy-related diabetes. * Different ethnicity-specific cutoff values for waist circumference have been proposed by the International Diabetes Federation (e.g., 94 cm for European men and 80 cm for European women) [5].

**Anthropometry**
a. BMI
b. Waist circumference *
c. Waist-to-hip ratio
d. Waist-to-height ratio
**Body composition**
a. Bioelectrical impedance: vector analysis, phase angle
b. Nutritional ultrasound
c. Liver ultrasound
d. Liver elastography
e. Others: plethysmography, DEXA, CT, MRI
**Muscle functionality**
a. Dynamometry
b. Sit-to-stand test
c. 6-min walk test
**Laboratory tests**
a. Metabolic syndrome
-HbA1c
-HOMA-IR
-Lipid profile
-C-reactive protein
-FIB-4 score
-Adipokines
-Urinary albumin/creatinine ratio
b. Beta-cell function
-Fasting plasma glucose, HbA1c, continuous glucose monitoring
-Basal and/or stimulated C-peptide
-HOMA-B
-Pancreatic autoimmunity
-HLA genotypes at risk for type 1 diabetes
**Differential Diagnosis with Other Types of Diabetes**
a. Tests for monogenic diabetes
-Online probability calculators
-Genetic testing
b. Tests for diabetes associated with pancreatic disease
-Fecal elastase
-Tumor markers
-Pancreatic imaging studies
c. Hormonal tests for endocrinopathies (e.g., Cushing’s disease, acromegaly)
d. Tests for hereditary hemochromatosis
-Transferrin saturation index
-Genetic testing

**Table 4:** Clinical trials demonstrating cardiovascular or renal benefits of currently available GLP-1 receptor agonists and SGLT-2 inhibitors for the treatment of patients with T2D. * Studies including individuals with and without T2D [24,98,99,100,101,102,103,104,105].

Drugs with CV Benefit in Specific Study Populations	Clinical Trial	Primary and Secondary Endpoints with a Significant Risk Reduction	
		Major CV Events and HF Hospitalization	Renal Outcome
**Established CVD or multiple CV risk factors**			
**GLP-1 receptor agonists**			
Liraglutide	LEADER	MACE3, mortality	Lower progression of CKD
Semaglutide (sc)	SUSTAIN-6	MACE3	Lower progression of CKD
Dulaglutide	REWIND	MACE3	Lower progression of CKD
Semaglutide (oral)	SOUL	MACE3	
**SGLT2 inhibitors**			
Empagliflozin	EMPA-REG OUTCOME	MACE3,mortality, HF hospitalization	Lower progression of CKD
Canagliflozin	CANVAS	MACE3, HF hospitalization	Lower progression of CKD
Dapagliflozin	DECLARE-TIMI	HF hospitalization	Lower progression of CKD
**Heart failure with reduced ejection fraction**			
**SGLT2 inhibitors**			
Dapagliflozin	DAPA-HF *	HF hospitalization, mortality	
Empagliflozin	EMPEROR-Reduced *	HF hospitalization	Lower progression of CKD
**Heart failure with preserved ejection fraction**			
**SGLT2 inhibitors**			
Empagliflozin	EMPEROR-Preserved *	HF hospitalization	
Dapagliflozin	DELIVER *	HF hospitalization	
**GLP-1 receptor agonists**			
Semaglutide 2.4 (sc)	STEP-HFpEF DM	HF clinical improvement	
**Dual GLP-1/GIP receptor agonists**			
Tirzepatide 15 mg	SUMMIT	HF hospitalization, HF clinical improvement	
**Chronic kidney disease with albuminuria**			
**SGLT2 inhibitors**			
Canagliflozin	CREDENCE	MACE3, HF hospitalization	Lower progression of CKD
Dapagliflozin	DAPA-CKD *	HF hospitalization, mortality	Lower progression of CKD
**GLP-1 receptor agonists**			
Semaglutide (sc)	FLOW	MACE3, mortality	Lower progression of CKD
**Chronic kidney disease with or without albuminuria**			
	**SGLT2 inhibitors**		
Empagliflozin	EMPA-KIDNEY *		Lower progression of CKD
